# Cromoglycate and Nedocromil: Influence on Airway Reactivity

**DOI:** 10.1155/S0962935194000694

**Published:** 1994

**Authors:** E. A. Valletta, A. L. Boner

**Affiliations:** 1 Paediatric Department Policlinico B. Roma University of Verona Verona I-37134 Italy; 2 Istituto Pio XII Misurina (BL) Italy

## Abstract

Although basic mechanisms of bronchial hyper-responsiveness (BHR) are still incompletely understood, inflammation of airways is likely to play a fundamental role in modulating BHR in patients with asthma. The involvement of several inflammatory cells (eosinophils, mast cells, lymphocytes, neutrophils, macrophages and platelets) and of bioactive mediators secreted by these cells in the pathogenesis of asthma is well documented. Sodium cromoglycate and nedocromil sodium are two pharmacological agents which have anti-allergic and anti-inflammatory properties. Their clinical effectiveness in mild to moderate asthma, and the capacity to reduce BHR under different natural and experimental conditions, make them valuable drugs for maintenance therapy in patients with asthma.


Mediators of Inflammation 3, S15–S19 (1994)

**ALTHOUGH basic mechanisms of bronchial hyper-responsiveness (BHR) are still incompletely understood, inflammation of airways is likely to play a fundamental role in modulating BHR in patients with asthma. The involvement of several inflammatory cells (eosinophils, mast cells, lymphocytes, neutrophils, macrophages and platelets) and of bioactive mediators secreted by these cells in the pathogenesis of asthma is well documented. Sodium cromoglycate and nedocromil sodium are two pharmacological agents which have anti-allergic and anti-inflammatory properties. Their clinical effectiveness in mild to moderate asthma, and the capacity to reduce BHR under different natural and experimental conditions, make them valuable drugs for maintenance therapy in patients with asthma.**

**Key words**: Airway reactivity, Bronchial hyper-responsiveness, Cromoglycate, Nedocromil

### Cromoglycate and nedocromil: influence on airway reactivity

E. A. Valletta and A. L. Boner[CA]

Paediatric Department, Policlinico B. Roma, University of Verona, I-37134 Verona, and Istituto Pio XII, Misurina (BL), Italy

[CA] Corresponding Author

### Bronchial Hyper-responsiveness

Bronchial hyper-responsiveness (BHR) is defined as an abnormal responsiveness of the airways which determines an increased air flow obstruction on exposure to different stimuli. Classically, these stimuli have been divided into specific stimuli such as allergens and chemicals, and non-specific stimuli such as histamine, methacholine, cold air and exercise. Nevertheless, this classification does not seem completely adequate since the so-called non-specific stimuli also act through different and specific mechanisms.[1] Although some authors feel that BHR is acquired during life,[2] there is evidence that constitutional[3] and familial or genetic factors[4] play a major role in predisposing to BHR. The risk of BHR is related to the degree and the severity of atopy.[5] In allergic asthmatics, BHR increases during prolonged exposition to inhaled allergens (e.g. during the pollen season) and significantly decreases after adequate environmental control.[6,7] There is also a strong association between the degree of BHR and asthma severity and medication requirement,[8] and BHR is considered to be a risk factor for the outcome of childhood asthma.[9]

Although the factors that may contribute to BHR are still incompletely known, there is today strong evidence that the inflammatory process that occurs in the airways in asthma is the key event in the modulation of BHR.[10] Studies on bronchial alveolar lavage (BAL), sputum or bronchial biopsies in subjects with asthma are all consistent with the involvement of eosinophils, mast cells, lymphocytes, neutrophils, macrophages and platelets in airway inflammation.[8] Consequently, a variety of inflammatory mediators are locally active, such as histamine, prostanoids, leukotrienes, kinins, eosinophil proteins, mast cell tryptase and neuropeptides.[11] The role of mast cells seems to be of the greatest importance in the initial phases of allergic inflammation. Two populations of these cells have so far been identified in human lungs. One is located near blood vessels and fibrous stroma, and can be studied only after enzymatic dissociation of whole lung tissue. The second population is between the basement membrane and the epithelium, and can be easily recovered in BAL. The two types of pulmonary mast cells show subtle functional differences, and they probably play distinct roles in the pathogenesis of asthma.[12,13] Because of their location, BAL mast cells would come into immediate contact with inhaled allergens and consequently might be involved in the initial events of the asthmatic response. The number of BAL mast cells is increased in subjects with asthma and it correlates with the degree of airway obstruction and hypersensitivity.[13]

The inflammatory response and mediator release might influence BHR through the increase of airway epithelial permeability, the decrease in airway calibre, and the modification of autonomic control or myogenic function.[2,14] Viral infections and ozone, which cause reversible damage of the airway epithelium, also result in a transient increase of bronchial reactivity.[15] Recently, it has been shown that inflammation also occurs in the airways of mildly asthmatic patients with normal pulmonary function but with BHR to histamine or methacholine.[16,17]

With respect to the ability to induce airway inflammation, the different stimuli can be divided into two groups.[18] The first group includes allergens, occupa-

© 1994 Rapid Communications of Oxford Ltd tional chemical substances (such as isocyanates), and certain viral infections which can cause both airway constriction and airway inflammation. The second group of stimuli includes histamine, methacholine, exercise, cold air and hypertonic solutions which are responsible for a substantial bronchoconstriction. Airway response to these stimuli is strongly influenced by the degree of pre-existent inflammation.

Allergen inhalation in subjects with asthma causes, at 10–15 min interval, an early asthmatic response which is frequently (70% or more in children) followed by a late bronchoconstriction that peaks at 6–12 h after the challenge.[19,20] This late asthmatic response (LAR) is strongly connected with the cellular phase of airway inflammation, and is particularly associated with an increase in bronchial reactivity to histamine and methacholine which may last several days or weeks.[21–23] We can assume that the increase of bronchial reactivity to histamine or methacholine indirectly reflects the degree of airway inflammation in asthma.[1]

Based on the concept that inflammatory processes are of paramount importance in modulating bronchial reactivity and clinical symptoms, in patients with chronic asthma it makes sense to use preferentially those drugs that treat the underlying disease because of their anti-inflammatory activity. Presently, there is sufficient evidence to suggest that $\beta_2$-agonists, anticholinergic drugs and xanthines have little, if any, anti-inflammatory activity, and that they have no significant long-term effects on airway reactivity.[24] On the other hand, drugs with an appreciable anti-inflammatory activity are already available (corticosteroids, sodium cromoglycate, nedocromil sodium) and other anti-inflammatory medications (cyclosporin, leukotriene antagonists, 5-lipoxygenase inhibitors, platelet activating factor receptor blockers) are being studied in the treatment of severe asthma.[25] In the following paragraphs, we will focus on the role of sodium cromoglycate and nedocromil sodium in inducing modifications of BHR and of the underlying inflammatory processes.

### Sodium Cromoglycate

Since its discovery, sodium cromoglycate (SCG) was found to have minimal bronchodilatory effect in both animal and human models, while it showed significant activity in modulating inflammatory events involved in the pathogenesis of allergic diseases. In the past 30 years a large amount of research has been devoted to the study of the biological and clinical effects of SCG. Nevertheless, the basic mechanisms of action of this drug are not completely understood.[26] At present, there is a reasonable consensus on at least some aspects of the activity of SCG (Table 1). The first and the most extensively studied mechanisms are the stabilization of mast cell membranes and the inhibition of histamine release during antigen challenge. Very high concentrations of SCG are required to prevent degranulation of mast cells from dispersed lung tissue,[27] while concentrations which are clinically achievable are effective in inhibiting BAL mast cell degranulation.[28] This effect on mast cell degranulation is thought to be dependent on two main molecular events: blockage of extracellular calcium shift into the cell and phosphorylation of specific membrane proteins.[29,30]

SCG is also believed to interfere with inflammatory processes in asthma by inhibiting the activation of important inflammatory cells, such as eosinophils and monocytes, and eosinophil chemotaxis induced by platelet activating factor.[31] Finally, prevention against capsaicin-substance P-induced bronchoconstriction in dogs and bronchospasm caused by sulphur dioxide, metabisulphites and isocyanates in human beings, are thought to be due to a blocking effect on neural reflex bronchoconstriction.[32]

The effectiveness of SCG in the treatment of childhood asthma has been well documented in several clinical studies. When administered regularly, it decreases respiratory symptom scores and the need for adjunctive anti-asthma medications such as theophylline, $\beta_2$-agonists and corticosteroids.[33] Improvement in asthma control by SCG is thought to be largely dependent on its capacity to modulate non-specific bronchial hyper-responsiveness at therapeutic doses.

For practical purposes, it is useful to analyse the studies on the effect of SCG on BHR by dividing them into short-term and long-term studies. In the former, acute protection is determined by the shift of the dose–response curve immediately after drug administration. In the latter, BHR is measured after several weeks (or months) of treatment.

**TABLE 1.** Mechanisms of action of sodium cromoglycate and nedocromil sodium

| |
|---|
| *Sodium cromoglycate* |
| Stabilization of mast cell membrane |
| Protein phosphorylation |
| Inhibition of the activation of inflammatory cells |
| Inhibition of the effects of cell mediators |
| Inhibition of neuropeptide-induced neural reflex bronchoconstriction |
| *Nedocromil sodium* |
| Stabilization of mast cell membrane |
| Inhibition of mediator release by inflammatory cells |
| Inhibition of neutrophil and eosinophil chemotaxis induced by PAF |
| Inhibition of protein kinase C |
| Inhibition of neuropeptide release from sensory nerves |

(Modified from: Bernstein JA and Berstein IL. Cromolyn and nedocromil. Novel anti-allergic drugs. *Immunol Allergy Clin N Am* 1993; **13**: 891–902.)

SCG inhibits both the early and late phase airway response when administered before allergen challenge[34] and this is associated with an inhibitory effect on the allergen-induced hyper-responsiveness.[34,35] Administration of SCG after the early response, but at least 60 min before the late response to allergen, caused a significant delay of LAR onset and a reduction in its duration.[36] Furthermore, the allergen-induced increase in methacholine responsiveness was also prevented.[36]

Short-term studies which evaluated the protective effect of SCG against nonantigenic bronchial challenges showed variable results, depending on the bronchoprovocative agent utilized. Acute protection of SCG (40 mg, 10 min before challenge) to methacholine and histamine was observed by Woenne *et al.*[37] in 60% of a group of children with asthma. Mean change in BHR was +1.3 doubling doses (DD) and 0.8 DD when tested by methacholine and histamine, respectively. On the other hand, Cockcroft *et al.*,[38] Griffin *et al.*[39] and Lemire *et al.*[40] failed to observe any significant protection of short-term SCG against BHR to histamine or methacholine.

SCG has a well-documented preventive effect against bronchoconstriction induced by exercise,[41,42] cold air[43] and nonisotonic aerosol inhalation.[44] SCG is also more effective than atropine in preventing the asthmatic response to sulphur dioxide which is mediated by atropine-sensitive mechanisms, probably through irritant airway receptors.[45]

Long-term studies substantially confirmed the protective effect of SCG against allergen inhalation challenge (2–4 weeks of administration)[46] and cold air (4 weeks).[39] In exercise-induced bronchoconstriction, the drug effectiveness was unaffected by its chronic use (1 year).[47] The results of chronic dosing of SCG upon histamine and methacholine challenge were conflicting when SCG was used for up to 8 weeks. Lack of activity was reported by some authors,[39,48–50] while positive results—especially for 6 to 8 week studies—were observed by other investigators.[51,52] Conflicting results disappear when SCG is used chronically for more than 8 weeks. In such a case, a significant reduction of BHR, as measured by histamine or methacholine challenge, has been reported by most authors.[33,47,53]

### Nedocromil sodium

Although nedocromil sodium (NS) differs structurally from SCG, they have many common mechanisms of action (Table 1). NS reduces both early and late airway responses to allergen challenge by inhibiting mediator release from a variety of inflammatory cells.[54] It inhibits histamine secretion from both human BAL and dispersed lung mast cells, but its apparent activity varies markedly and inversely with the strength of the secretory stimulus.[55] NS also inhibits the release of $PGD_2$ from human lung mast cells[56] and strongly decreases *in vitro* neutrophil and eosinophil mobilization caused by different chemotactic factors such as PAF and $LTB_4$.[57] In an *in vitro* model of human bronchial tissue, NS inhibited the hyper-responsiveness induced by ionophore activated neutrophils presumably by modulating the release of prostaglandins and/or thromboxane $A_2$ from inflammatory cells.[58] NS also induces a significant inhibition of generation of cytotoxic mediators from both human monocytes and alveolar macrophages[59] and of the release of leukotrienes and 5-HETE from alveolar macrophages.[60] In human neutrophils, a dose-dependent interference with *N*-formyl-methionyl-leucyl-phenylalanine (fMLP)-induced superoxide anion production and with fMLP binding was observed.[61,62] Finally, the inhibition of bronchoconstriction induced by inhaled bradykinin sulphur dioxide, metabisulphite and ultrasonically nebulized water[63] suggests that NS prevents sensitization and activation of airway sensory nerves.

Clinical studies on the use of NS in patients with asthma have generally demonstrated a beneficial effect on respiratory symptoms and function similar to those obtained with SCG. Single-dose studies confirmed that NS significantly protects against allergen, fog and exercise challenge.[64–68] In exercise-induced bronchoconstriction in children, NS is more effective than the calcium antagonists verapamil and ipratropium bromide, but is comparable with SCG.[68] NS seems to offer a better prevention than SCG against inhaled adenosine[69], sulphur dioxide[70] and cold air.[43] Recently, it was shown that in nonasthmatic, nonatopic subjects, NS administration for 4 days significantly inhibited a PAF-induced increase in airway reactivity to methacholine.[71] Long-term (8 weeks) treatment with NS or beclomethasone resulted in a comparable decrease of BHR to methacholine in nonatopic asthmatic adults[72] but, in another study, an 8-week treatment with beclomethasone was reported to be superior to treatment with NS with regard to BHR to histamine and distilled water in atopic asthmatic adults.[73] A decrease in histamine responsiveness was also observed when NS was used for 4 weeks during seasonal allergen exposure[74] but no change was reported in 120 clinically stable asthmatic children, when challenged after NS administration for 8 weeks,[75] despite improving pulmonary function in those with abnormal baseline levels. Finally, experimental data suggest that NS and beclomethasone probably reduce BHR by different mechanisms.[72] This view is supported by the fact that addition of NS to inhaled corticosteroids can improve clinical symptoms and respiratory function[76,77] in steroid-dependent adult patients.

### Conclusions

Airway inflammation and the release of mediators from inflammatory cells have recently received attention as important factors in the pathogenesis of BHR in asthmatic patients. Consequently, a wider use of antiallergic drugs for the long-term treatment of asthma seems advisable. Although there is increasing recognition that BHR and asthma are not synonymous, the capacity of a drug to reduce BHR under different natural and experimental conditions can be still considered a useful method to explore its clinical efficacy. SCG and NS demonstrated, both *in vitro* and *in vivo*, a good antiallergic/anti-inflammatory activity and they offer protection against various inhalation challenges, both immunologic and nonimmunologic. The results of the available studies suggest that SCG and NS can be safely considered in the choice of a first-line therapy for the maintenance treatment of mild-to-moderate asthma.

### References

1. Hargreave FE, Gibson PG, Ramsdale EH. Airway hyper-responsiveness, airway inflammation, and asthma. *Immunol Allergy Clin N Am* 1990; **10**: 439–448.
2. Hargreave FE, Dolovich J, O'Byrne PM, *et al.* The origin of airway hyper-responsiveness. *J Allergy Clin Immunol* 1986; **78**: 825–832.
3. Martinez FD, Morgan WJ, Wright AL, *et al.* Diminished lung function as a predisposing factor for wheezing respiratory illness in infants. *N Engl J Med* 1988; **319**: 1112–1117.
4. Young S, Le Souef PN, Geelhoed GC, *et al.* The influence of a family history of asthma and parental smoking on airway responsiveness in early infancy. *N Engl J Med* 1991; **324**: 1168–1173.
5. Peat JK, Britton WJ, Salome CM, *et al.* Bronchial hyper-responsiveness in two populations of Australian schoolchildren. II. Relative importance of associated factors. *Clin Allergy* 1987; **17**: 283–290.
6. Platt-Mills TAE, Tovey ER, Mitchell EB, *et al.* Reduction of bronchial hyper-reactivity during prolonged allergen avoidance. *Lancet* 1982; **ii**: 675–678.
7. Boner AL, Niero E, Antolini I, *et al.* Pulmonary function and bronchial hyper-reactivity in asthmatic children with house dust mite allergy during prolonged stay in the Italian Alps (Misurina, 1756 m). *Ann Allergy* 1985; **54**: 42–45.
8. Scholsberg M, Liu MC, Bochner BS. Pathophysiology of asthma. *Immunol Allergy Clin N Am* 1993; **13**: 721–743.
9. Martin AJ, Landau LI, Phelan PD. Lung function in young adults who had asthma in childhood. *Am Rev Respir Dis* 1980; **122**: 609–616.
10. Pattemore PK, Holgate ST. Bronchial hyper-responsiveness and its relationship to asthma in childhood. *Clin Exp Allergy* 1993; **23**: 886–900.
11. Djukanovic R, Roche WR, Wilson JW, *et al.* Mucosal inflammation in asthma. *Am Rev Respir Dis* 1990; **142**: 434–457.
12. Pearce FL, Flint KC, Leung KBP, *et al.* Some studies on human pulmonary mast cells obtained by bronchoalveolar lavage and by enzymic dissociation of whole lung tissue. *Int Arch Allergy Appl Immunol* 1987; **82**: 507–512.
13. Pearce FL. Effect of nedocromil sodium on mediator release from mast cells. *J Allergy Clin Immunol* 1993; **92**: 155–158.
14. Eggleston PA. Upper airway inflammatory diseases and bronchial hyper-responsiveness. *J Allergy Clin Immunol* 1988; **81**: 1036–1041.
15. Boushey HA, Holtzman MJ, Sheller JR, Nadel JA. Bronchial hyper-reactivity. *Am Rev Respir Dis* 1980; **121**: 389–413.
16. Lozewicz S, Gomez E, Ferguson H, *et al.* Inflammatory cells in the airways in mild asthma. *Br Med J* 1988; **297**: 1515–1516.
17. Beasley R, Roche WR, Roberts JA, *et al.* Cellular events in the bronchi in mild asthma and after bronchial provocation. *Am Rev Respir Dis* 1989; **139**: 806–817.
18. Dolovich J, Hargreave FE. The asthma syndrome: inciters, inducers and host characteristics. *Thorax* 1981; **36**: 641–644.
19. Warner JO. Significance of late reactions after bronchial challenge with house dust mite. *Arch Dis Childhood* 1976; **51**: 905–910.
20. Robertson DG, Kerigan AT, Hargreave FE, Dolovich J. Late asthmatic responses induced by ragweed pollen allergen. *J Allergy Clin Immunol* 1974; **54**: 244–254.
21. Durham SR. Late onset reactions. In: Morley J, ed. *Preventive Therapy in Asthma.* London: Academic Press, 1991: 131–140.
22. O'Byrne PM, Dolovich J, Hargreave FE. Late asthmatic responses. *Am Rev Respir Dis* 1987; **136**: 740–751.
23. Virant FS, Bierman CW. The role of the neutrophil in the late-phase asthmatic reaction and airway hyper-responsiveness. *Immunol Allergy Clin N Am* 1990; **10**: 283–293.
24. van Essen-Zandvliet EEM, Kerrebijn KF. The effect of antiasthma drugs on bronchial hyper-responsiveness. *Immunol Allergy Clin N Am* 1990; **10**: 483–501.
25. Kaliner MA. How the current understanding of the pathophysiology of asthma influences our approach to therapy. *J Allergy Clin Immunol* 1993; **92**: 144–147.
26. Bernstein JA, Bernstein IL. Cromolyn and nedocromil: novel anti-allergic drugs. *Immunol Allergy Clin N Am* 1993; **13**: 891–902.
27. Church MK, Young KO. The characteristics of inhibition of histamine release from human lung fragments by sodium cromoglycate, salbutamol, and chlorpromazine. *Br J Pharmacol* 1983; **78**: 671–679.
28. Flint KC, Leung KBP, Pearce FL, *et al.* Human mast cells recovered by bronchoalveolar lavage: their morphology, histamine release and the effects of sodium cromoglycate. *Clin Sci* 1985; **68**: 427–432.
29. Mazurek N, Berger G, Pecht I. A binding site on mast cells and basophils for the antiallergic drug disodium cromoglycate. *Nature* 1980; **286**: 722–723.
30. Wells E, Mann J. Phosphorylation of a mast cell protein in response to treatment with anti-allergic compounds. *Biochem Pharmacol* 1983; **32**: 837–842.
31. Basran GS, Page CT, Paul W, *et al.* Cromoglycate inhibits the responses to plate-activating factor (PAF-acether) in man: an alternative mode of action for DSCG in asthma? *Eur J Pharmacol* 1983; **86**: 143–147.
32. Harries MG. Bronchial irritant receptors and a possible new action for cromolyn sodium. *Ann Allergy* 1981; **46**: 156–158.
33. Furukawa CT, Shapiro GG, Bierman CW, *et al.* A double-blind study comparing the effectiveness of cromolyn sodium and sustained release theophylline in childhood asthma. *Pediatrics* 1984; **74**: 453–459.
34. Cockcroft DW, Murdock KY. Comparative effects of inhaled salbutamol sodium cromoglycate and beclomethasone dipropionate on allergen-induced early asthmatic response, late asthmatic response and allergen-induced increases in bronchial responsiveness to histamine. *J Allergy Clin Immunol* 1987; **79**: 734–740.
35. Mattoli S, Foresi A, Corbo GM, *et al.* Protective effect of disodium cromoglycate on allergen-induced bronchoconstriction and increased hyper-responsiveness: a double-blind placebo-controlled study. *Ann Allergy* 1986; **57**: 295–300.
36. Mattoli S, Foresi A, Corbo GM, *et al.* Effects of two doses of cromolyn on allergen-induced late asthmatic response and increased responsiveness. *J Allergy Clin Immunol* 1987; **79**: 747–754.
37. Woenne R, Kattan M, Levison H. Sodium cromoglycate-induced changes in the dose-response curve of inhaled methacholine and histamine in asthmatic children. *Am Rev Respir Dis* 1979; **119**: 927–930.
38. Cockcroft DW, Kilian DN, Mellon JJA, Hargreave FE. Protective effect of drugs on histamine-induced asthma. *Thorax* 1977; **32**: 429–437.
39. Griffin MP, MacDonald N, McFadden ER. Short- and long-term effects of cromolyn sodium on the airway reactivity of asthmatics. *J Allergy Clin Immunol* 1983; **71**: 331–338.
40. Lemire I, Cartier A, Malo J-L, *et al.* Effect of sodium cromoglycate on histamine inhalation tests. *J Allergy Clin Immunol* 1984; **73**: 234–239.
41. Boner AL, Niero E, Grigolini C, *et al.* Inhibition of exercise-induced asthma by three forms of sodium cromoglycate. *Eur J Respir Dis* 1985; **66**: 21–24.
42. Davies SE. Effect of disodium cromoglycate on exercise-induced asthma. *Br Med J* 1968; **3**: 593–594.
43. del Bono L, Dente FL, Patalano F, del Bono N. Protective effect of nedocromil sodium and sodium cromoglycate on bronchospasm induced by cold air. *Eur J Respir Dis* 1986; **6** (Suppl 147): 268–270.
44. Anderson SD. Bronchial challenge by ultrasonically nebulized aerosols. *Clin Rev Allergy* 1985; **3**: 427–439.
45. Koenig JQ, Marshall SG, van Belle G, *et al.* Therapeutic range cromolyn dose-response inhibition and complete obliteration of SO2-induced bronchoconstriction in atopic adolescents. *J Allergy Clin Immunol* 1988; **81**: 897–901.
46. Ryo UY, Kang B, Townley RG. Effect of disodium cromoglycate on inhalation challenge with allergen, histamine and methacholine in subjects with bronchial asthma. *J Allergy* 1971; **47**: 96.
47. Dickson W. A one year's trial of Intal compound in 24 children with severe asthma. In: Pepys J, Frankland AW, eds. *Disodium Cromoglycate in Allergic Airway Disease.* London; Butterworths, 1970: 105.
48. Laitenen LA, Venho K, Poppius H. A controlled study on the effect of treatment with cromolyn sodium pressurized aerosol on bronchial reactivity in patients with asthma. *Ann Allergy* 1986; **56**: 270–273.
49. Lowhagen O, Rak S. Bronchial hyper-reactivity after treatment with sodium cromoglycate in atopic asthmatic patients not exposed to relevant allergens. *J Allergy Clin Immunol* 1985; **75**: 343–347.
50. Svendsen UG, Frolund L, Madsen F, *et al.* A comparison of the effects of sodium cromoglycate and beclomethasone dipropionate on pulmonary function and bronchial hyper-reactivity in subjects with asthma. *J Allergy Clin Immunol* 1987; **80**: 68–74.
51. Lowhagen O, Rak S. Effect of sodium cromoglycate and budesonide on bronchial hyper-reactivity in non-atopic asthmatics. *Respiration* 1984; **46** (Suppl 1): 105.
52. Lowhagen O, Rak S. Modification of bronchial hyper-reactivity after treatment with sodium cromoglycate during pollen season. *J Allergy Clin Immunol* 1985; **75**: 460–467.
53. Petty T, Rollins LD, Christopher K, *et al.* Cromolyn sodium is effective in adult chronic asthmatics. *Am Rev Respir Dis* 1989; **139**: 694–701.
54. Abraham WM, Stevenson JS, Chapman GA, *et al.* The effect of nedocromil sodium and cromolyn sodium on antigen-induced responses in allergic sheep *in vivo* and *in vitro.* 1987; **92**: 913–917.
55. Leung KBP, Flint FC, Brostoff J, *et al.* Effects of sodium cromoglycate and nedocromil sodium on histamine secretion from human lung mast cells. *Thorax* 1988; **43**: 756–761.
56. Flint KC, Leung KBP, Hudspith BN, *et al.* Bronchoalveolar mast cells in extrinsic asthma: a mechanism for the initiation of antigen-specific bronchoconstriction. *Br Med J* 1985; **291**: 923–926.

57. Bruijnzel PLB, Warringa RAJ, Kok TM, *et al.* Effects of nedocromil sodium on *in vitro* induced migration, activation and mediator release from human granulocytes. *J Allergy Clin Immunol* 1993; **92**: 159–164.
58. Hughes JM, McKay KO, Johnson PR, *et al.* Neutrophil-induced human bronchial hyper-responsiveness *in vitro*-pharmacological modulation. *Clin Exp Allergy* 1993; **23**: 251–256.
59. Joseph M, Tsicopoulos A, Tonnel A-B, Capron A. Modulation by nedocromil sodium of immunologic and nonimmunologic activation of monocytes, macrophages and platelets. *J Allergy Clin Immunol* 1993; **92**: 165–170.
60. Gonzalez JP, Brodgen RN. Nedocromil sodium: a preliminary review of its pharmacodynamic and pharmacokinetic properties, and therapeutic efficacy in the treatment of reversible obstructive airway disease. *Drugs* 1987; **334**: 560–577.
61. Peroni DG, Melotti P, Piacentini GL, *et al.* Effects of nedocromil sodium on the binding of *N*-formyl-methionyl-leucyl-phenylalanine in human neutrophils. *Agents Action* 1992; **36**: 212–214.
62. Peroni DG, Piacentini GL, Melotti P, Boner AL. Inhibition by nedocromil sodium of superoxide anion production in human neutrophils. A preliminary communication. *Drug Invest* 1992; **4**: 386–390.
63. Barnes PJ. Effect of nedocromil sodium on airway sensory nerves. *J Allergy Clin Immunol* 1993; **92**: 182–186.
64. Nair N, Hopp R, Townley R. Effect of nedocromil on antigen-induced bronchoconstriction in asthmatic subjects. *Ann Allergy* 1989; **62**: 329–331.
65. Debelic M. Nedocromil sodium and exercise-induced asthma in adolescents. *Eur J Respir Dis* 1986; **69** (Suppl 147): 266–267.
66. Robuschi M, Vaghi A, Simone P, *et al.* Prevention of fog-induced bronchospasm by nedocromil sodium. *Clin Allergy* 1987; **17**: 69–74.
67. Boner AL, Vallone G, Bennati D. Nedocromil sodium in exercise-induced bronchoconstriction in children. *Ann Allergy* 1989; **62**: 38–41.
68. Comis A, Valletta EA, Sette L, *et al.* Comparison of nedocromil sodium and sodium cromoglycate administered by pressurized aerosol, with and without a spacer device in exercise-induced asthma in children. *Eur Respir J* 1993; **6**: 523–526.
69. Crimi N, Palermo F, Olivieri R, *et al.* Adenosine-induced bronchoconstriction: comparison between nedocromil and sodium cromoglycate. *Eur J Respir Dis* 1986; **69** (Suppl 147): 258–262.
70. Altounyan REC, Cole M, Lee TB. Inhibition of sulphur dioxide induced bronchoconstriction by nedocromil sodium and sodium cromoglycate in non-asthmatic atopic subjects. *Eur J Resp Dis* 1986; **69** (Suppl 147): 274–276.
71. Di Maria GU, Bellofiore S, Ciancio N, *et al.* Nedocromil sodium inhibits the increase in airway reactivity induced by platelet activating factor in humans. *Chest* 1992; **102**: 123–128.
72. Bel EH, Timmers MC, Hermans J, *et al.* The long-term effects of nedocromil sodium and beclomethasone dipropionate on bronchial responsiveness to methacholine in nonatopic asthmatic subjects. *Am Rev Respir Dis* 1990; **141**: 21–28.
73. Groot CAR, Lammers J-WJ, Molema J, *et al.* Effect of inhaled beclomethasone and nedocromil sodium on bronchial hyper-responsiveness to histamine and distilled water. *Eur Respir J* 1992; **5**: 1075–1082.
74. Dorward AJ, Roberts JA, Thomson NC. Effect of nedocromil sodium on histamine airway responsiveness in grass-pollen sensitive asthmatics during the pollen season. *Clin Allergy* 1986; **16**: 309–316.
75. Foo AL, Lanteri CJ, Burton PR, *et al.* The effect of nedocromil sodium on histamine responsiveness in clinically stable asthmatic children. *J Asthma* 1993; **30**: 381–390.
76. Bianco S, del Bono N, Grassi V, Orefice U. Effectiveness of nedocromil sodium versus placebo as additions to routine asthma maintenance therapy: a multicentre, double-blind, placebo-controlled study. *Thorax* 1989; **44**: 654–659.
77. Rebuck AS, Kesten S, Boulet LP, *et al.* A 3-month evaluation of the efficacy of nedocromil sodium in asthma: a randomized, double-blind, placebo-controlled trial of nedocromil sodium conducted by a Canadian multicenter study group. *J Allergy Clin Immunol* 1990; **85**: 612–617.
